# Biomechanical changes in the proximal femur before and after removal of femoral neck system

**DOI:** 10.1186/s13018-024-04769-x

**Published:** 2024-05-12

**Authors:** Chong Nan, Yuxiu Liu, Di Zhang, Yazhuo Qin, Hetong Yu, Zhanbei Ma

**Affiliations:** 1 Department of Orthopedic, Baoding No. 1 Central Hospital, Baoding, Hebei Province 071000 China; 2 Department of Spinal Surgery, The Third Hospital of Hebei Medical University, Shijiazhuang, Hebei Province 050000 China

**Keywords:** Femoral neck system, Cannulated screw removal, Biomechanical effect, Finite element analysis

## Abstract

**Background:**

As an innovative internal fixation system, FNS (femoral neck system) is increasingly being utilized by surgeons for the treatment of femoral neck fractures. At present, there have been numerous finite element analysis experiments studying the immediate stability of FNS and CSS in treating femoral neck fractures. However, there is scarce mechanical analysis available regarding the effects post internal fixation removal. This study aimed to investigate the alterations in mechanical parameters of the proximal femur before and after the removal of FNS (femoral neck system), and to assess potential distinctions in indicators following the extraction of CSS (Cannulated Screws).

**Methods:**

A proximal femur model was reconstructed using finite element numerical techniques. The models for CSS and FNS were formulated utilizing characteristics and parametric definitions. The internal fixation was combined with a normal proximal femur model to simulate the healing state after fracture surgery. Within the framework of static analysis, consistent stress burdens were applied across the entirety of the models. The total deformation and equivalent stress of the proximal femur were recorded before and after the removal of internal fixation.

**Results:**

Under the standing condition, the total deformation of the model before and after removing CSS was 0.99 mm and 1.10 mm, respectively, indicating an increase of 12%. The total deformation of the model before and after removing FNS was 0.65 mm and 0.76 mm, respectively, indicating an increase of 17%. The equivalent stress for CSS and FNS were 55.21 MPa and 250.67 MPa, respectively. The average equivalent stress on the cross-section of the femoral neck before and after removal of CSS was 7.76 MPa and 6.11 MPa, respectively. The average equivalent stress on the cross-section of the femoral neck before and after removal of FNS was 9.89 MPa and 8.79 MPa, respectively.

**Conclusions:**

The retention of internal fixation may contribute to improved stability of the proximal femur. However, there still existed risks of stress concentration in internal fixation and stress shielding in the proximal femur. Compared to CSS, the removal of FNS results in larger bone tunnels and insufficient model stability. Further clinical interventions are recommended to address this issue.

## Background

As an innovative internal fixation system, FNS (femoral neck system) is increasingly being utilized by surgeons for the treatment of femoral neck fractures [1]. FNS offers angular stability through a combination of pressurization and anti-rotation properties. Numerous scholars have examined the biomechanical characteristics of FNS and refined the fixation technique. Jung [2] demonstrated that a lower positioning of the FNS bolt increases the glide distance between components, as well as the compression and shear stresses. He recommended a 5 mm distance between the plate and the bone. For unstable fractures, it has been suggested that the FNS be inserted in a lower position and combined with the application of a cannulated screw to prevent rotation [3]. When the angle of the fracture line exceeds 70°, some scholars recommend using FNS with a double-hole plate [4]. Correspondingly, the clinical efficacy of femoral neck fractures treated with FNS has been further elucidated. In Tang’s study, intraoperative bleeding and fluoroscopy times were significantly lower in the CCS (Cannulated Screws) group compared to the FNS group [5]. Patients in the FNS group experienced earlier weight-bearing and fracture healing times, lower rates of internal fixation failure, and better maintenance of femoral neck length compared to conventional CCS. These factors facilitated post-surgery recovery of hip function [6].

Meanwhile, controversy remains regarding the removal of internal fixations after radiological fracture healing. Some hypotheses suggested that this would lead to or exacerbate aseptic necrosis of the femoral neck and head. Jin established a predictive model for femoral head necrosis following femoral neck fracture surgery, with data indicating that removal of internal fixation is an independent risk factor [7]. Some scholars [8] speculated that the presence of internal fixation increased pressure within the femoral head, exacerbating damage to the femur. However, predicting the blood supply to the femoral head remains uncertain. Indeed, the decision to remove internal fixation necessitates a thorough assessment, encompassing factors such as patient age, subjective preferences, and potential complications. Some researchers conducted mechanical experiments to study the mechanical characteristics after removing hollow screws. In vitro experiments [9] indicated that removing hollow screws did not alter the maximum load of subsequent femoral neck fractures. Finite element experiments [10] further demonstrated that retained screws exhibited stress concentration and stress shielding effects. To our knowledge, there is currently limited research on mechanical experiments conducted after the removal of FNS.

Therefore, finite element numerical techniques were utilized to reconstruct CSS and FNS models. Conducting in vitro cadaver experiments requires significant resources including time, money, and manpower. In contrast, finite element analysis is typically more economical as it only requires a computer and software. Finite element analysis allows users to easily modify and adjust models to test different conditions and hypotheses without the need to set up experiments anew. The objective was to investigate the alterations in mechanical parameters of the proximal femur before and after the removal of FNS, and to assess potential distinctions in indicators following the extraction of various internal fixation devices.

## Materials and methods

### An illustrative clinical case

**Fig. 1:**
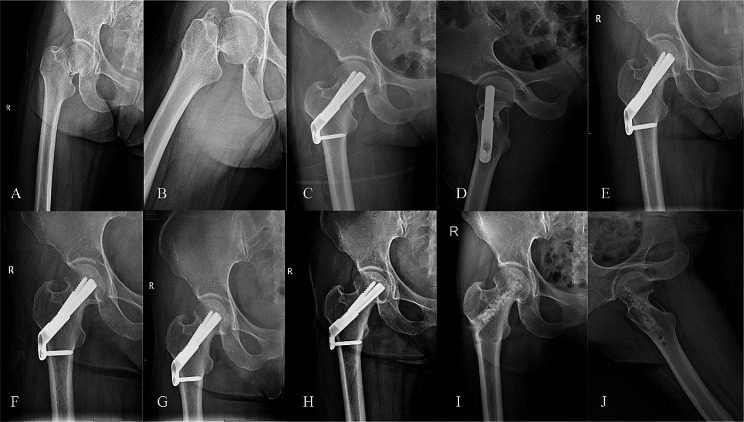
Radiographic images of a typical clinical case: X-ray during preoperative examination (**A**, **B**); Placement of FNS after closed reduction (**C**, **D**); X-ray at 3 months postoperatively (**E**); X-ray at 6 months postoperatively (**F**); X-ray at 12 months postoperatively (**G**); X-ray at 18 months postoperatively (**H**); Bone grafting performed after removal of internal fixation (**I**,**J**)

One instance of a 49-year-old healthy female diagnosed with a femoral neck injury resulting from a traffic accident(Fig. 1A, B). Completion of preoperative examinations followed by femoral neck fracture reduction and internal fixation with FNS placement(Fig. 1C, D). The patient underwent regular follow-up appointments at the orthopedic outpatient clinic for X-ray examinations(Fig. 1E-H). After fracture healing, the patient voluntarily requested removal of the internal fixation. After complete removal of internal fixation, bone grafting was performed in the nail canal(Fig. 1I, J). The patient has been under continuous follow-up for 5 years and is currently leading a normal life with regular work activities.

### Building the initial model

The recruited volunteer (60 years old) had no history of hip or systemic diseases. Written informed consent was obtained before the study. The subject’s normal proximal femur was scanned using a computed tomography (CT) scanner, and the resulting images were imported as DICOM files into Mimics 21.0. Subsequently, the complete DICOM image was imported into Mimics 17.0, where viewing windows (Right, Left, Anterior, Posterior) were configured, and the CT Bone Segmentation function was utilized to isolate the bony structure. Due to inaccuracies in the CT scan and the presence of artifacts, some tissues were not adequately separated, necessitating further editing of the Mask. Initial smoothing of the model was performed using the Smooth function to generate a 3D model. However, due to the irregular shape of the femur, the model still exhibited roughness even after the initial smoothing, thus requiring exportation to Geomagic software (3DSystems Inc, Rock Hill, SC, USA) for additional refinement.

We initiated the process by employing the ‘Remove pegs’ command to initially smooth the surface, gradually transitioning to the ‘Remove features’ command to handle the global model surface components. Additionally, for areas with lesser curvature, we utilized commands such as ‘Carve knife’ and ‘Curvature deformation’. In regions exhibiting greater curvature, such as the femoral neck, it became imperative to eliminate local material to execute the ‘single hole fill’ function. Subsequently, we employed the ‘Mesh Doctor’ tool to assess surface quality and execute polygonal operations. Following adjustments to the surface sheets, a construction grid was applied, and intersecting areas were meticulously repaired to ensure geometric integrity. Finally, the surface underwent fitting, and a thorough deviation analysis was conducted. Based on the model in the published paper [10, 11], we performed the CT gray value assignment method to optimize the simulation of the proximal femur bone. Following the completion of fitting the surfaced model, it underwent verification before being imported into SolidWorks 2017(Dassault, France).

### Establishing internal fixation and postoperative femoral models

**Fig. 2:**
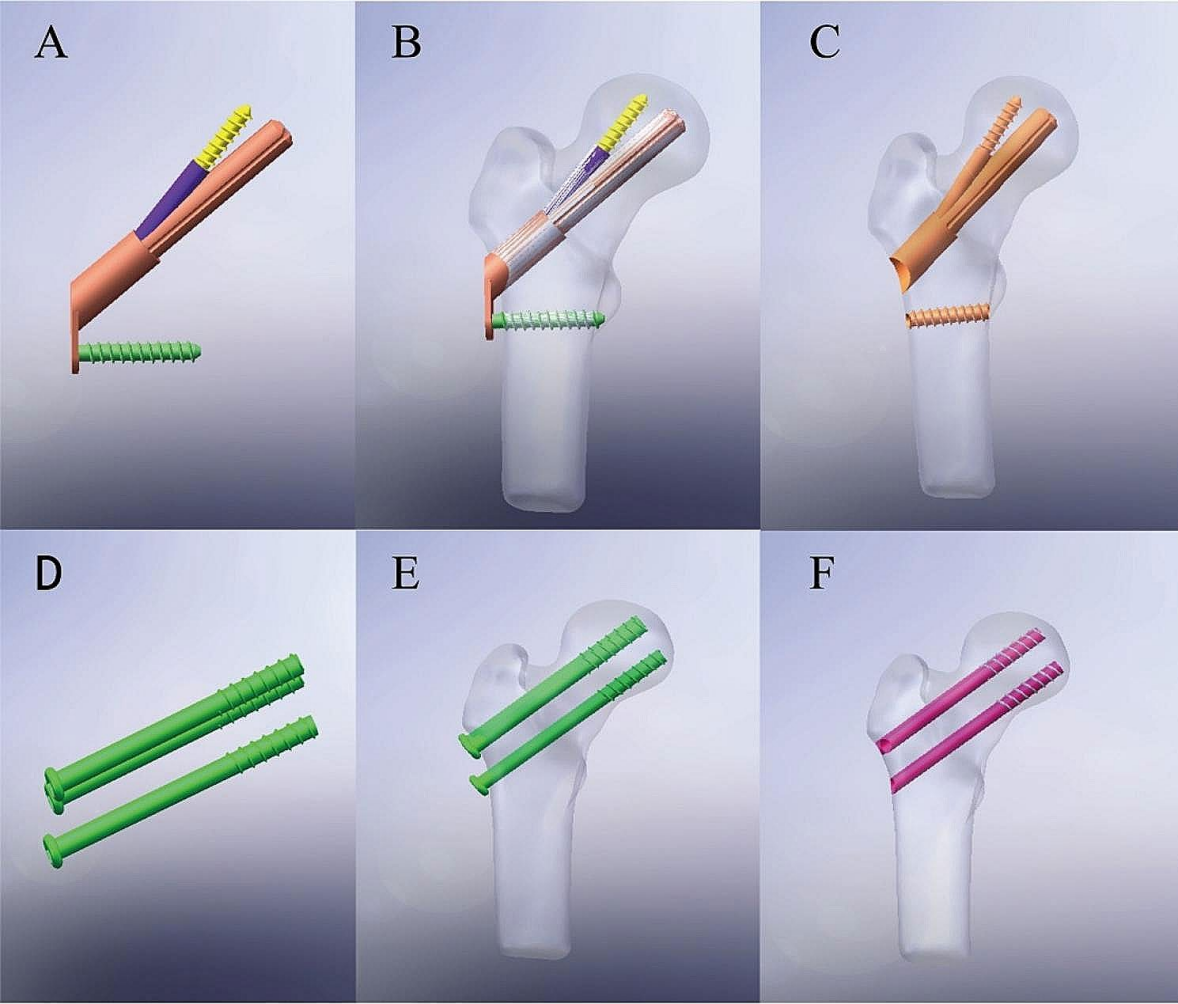
Conceptual diagrams of all models: FNS model (**A**); Preservation of FNS model (**B**);Removal of FNS model (**C**); CSS model (**D**); Preservation of CSS model (**E**); Removal of CSS model (**F**)

The models for the FNS and the cannulated cancellous screws were created utilizing a digital drawing application in SolidWorks. In the ‘assembly’ mode, the proximal femur and internal fixation were combined separately to simulate the model after fracture healing. The three cannulated screws(7.3 mm) were positioned parallel to the neck shaft angle (approximately 129 degrees) and arranged in a triangular pattern. The main nail of the FNS measured 85 mm in length and was positioned centrally along the coronal plane of the femoral neck, with a placement depth of 5 mm below the cartilage of the femoral head. Using the ‘direct editing’ command, entities were copied and combined using Boolean logic operations. The assembled components must undergo interference checking to avoid errors during post-processing in Ansys 17.0(Ansys, Canonsburg, PA, USA)(Fig. 2).

### Parameter setup and static structural analysis

The implant was presumed to be constructed from titanium alloy (Ti6Al4V), boasting a modulus of elasticity of 110 GPa and a Poisson’s ratio of 0.3. The contact conditions between the internal fixation screws and the femur were configured to simulate a bonded interface. Based on convergence experiments, a mesh size of 1 mm was selected. The farthest portion of the femur was stabilized, with limitations imposed on its range of motion. The conditions set for single-leg standing involved a force exerted on the hip joint that was roughly equivalent to threefold the individual’s body mass, which translates to a measurement of 1800 N [12]. The stress on the proximal femur was simplified, considering both the direction of force and muscle modeling [13, 14]. Finally, in the condition for single-leg standing, deformation and equivalent stress of the model were observed. Additionally, we applied a torsional load of 2.5 N·m to the femoral head to simulate twisting motions and recorded the total deformation of the model [15].

### Validation of the model’s efficacy

We compared our results with published finite element studies and in vitro experimental findings. The axial stiffness of our original model was 0.762 kN/mm, which closely approximated the reported 0.757 ± 0.264 kN/mm from the in vitro experiment [16]. The maximum strain value of the initial model was 1.258e-003 μm/N, which was similar to the findings in Zohar’s study(1.525e-003 μm/N) [17]. The initial model’s maximum equivalent stress was 17.05Mpa, similar to the findings in San’s study(17.48-18.05Mpa) [18]. Considering individual variations among models, the model established in this experiment is deemed effective.

## Results

**Fig. 3:**
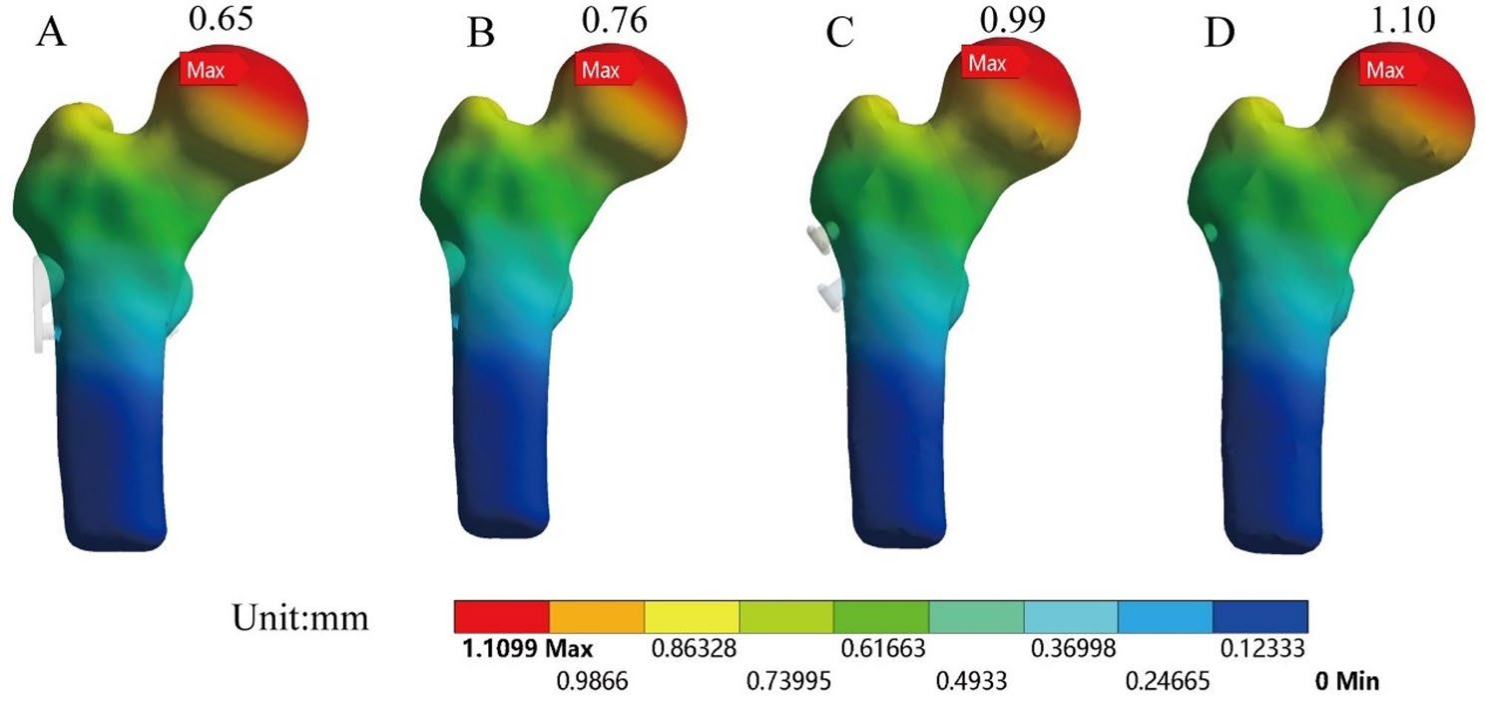
The total deformation of models: Preservation of FNS model (**A**); Removal of FNS model (**B**); Preservation of CSS model (**C**); Removal of CSS model (**D**)

**Fig. 4:**
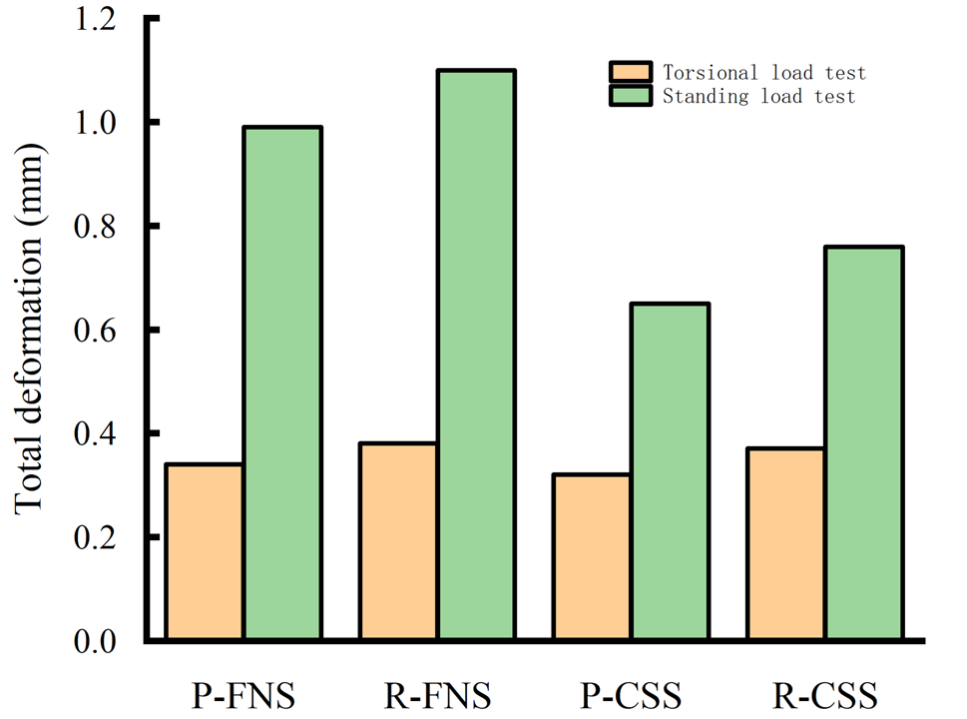
The total deformation of the model under torsional and standing conditions: P-FNS (Preservation of FNS model); R-FNS (Removal of FNS model); P-CSS (Preservation of CSS model); R-CSS (Removal of CSS model)

Figure 3 depicted the total deformation of each model before and after the removal of internal fixation. Figure 4 illustrated the total deformation under standing and torsional conditions. Total deformation can be used to assess the stability of structures under loading conditions. Larger total deformation may indicate excessive stress on the structure or the occurrence of instability, which could lead to structural failure or collapse. The red areas represented the regions of maximum deformation, with deformation in all models primarily concentrated in the weight-bearing region of the femoral head. The total deformation of the model before and after removing CSS was 0.99 mm and 1.10 mm, respectively, indicating an increase of 12%. The total deformation of the model before and after removing FNS was 0.65 mm and 0.76 mm, respectively, indicating an increase of 17%.

**Fig. 5:**
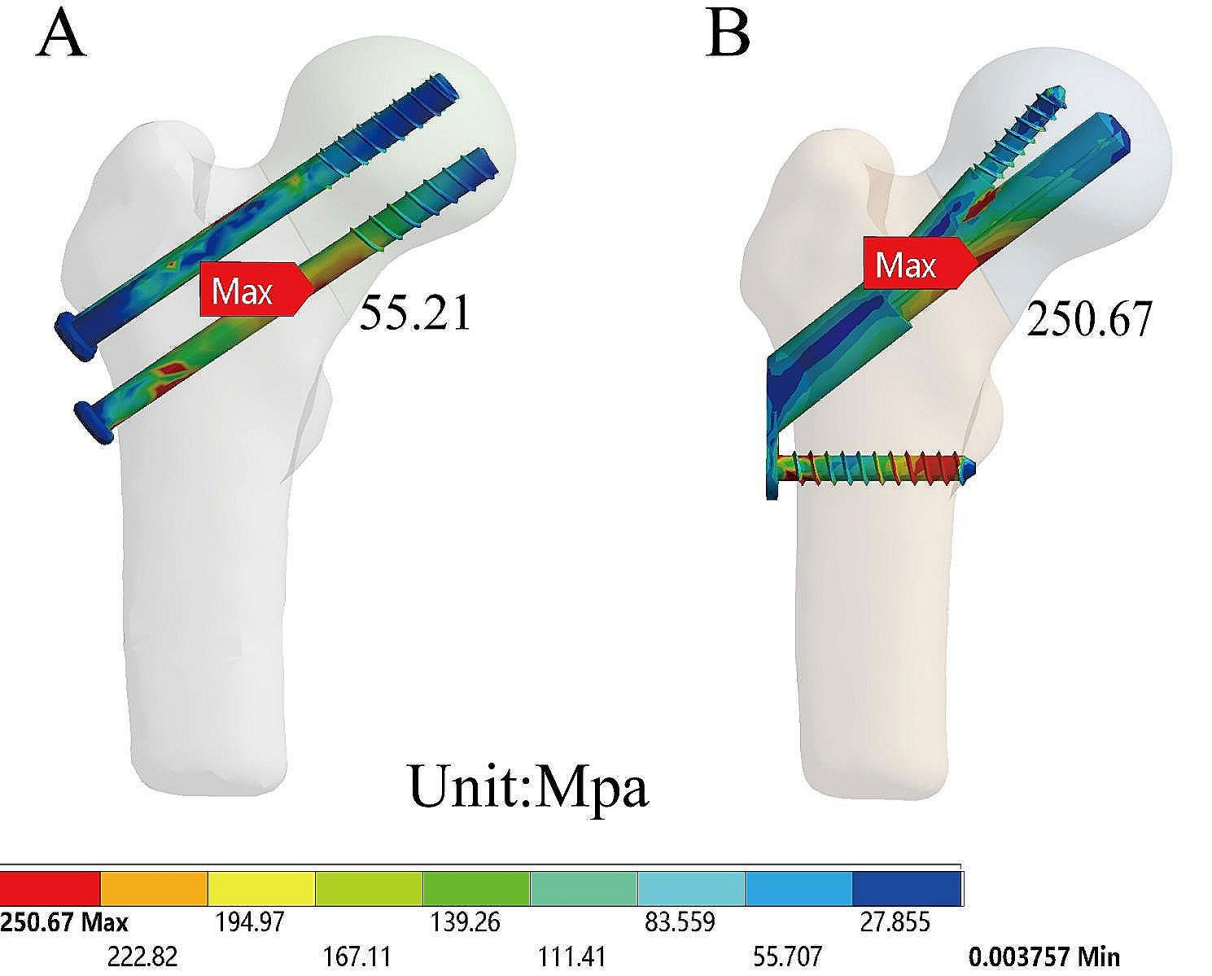
The equivalent stress of models: Preservation of CSS model (**A**); Preservation of FNS model (**B**)

**Fig. 6:**
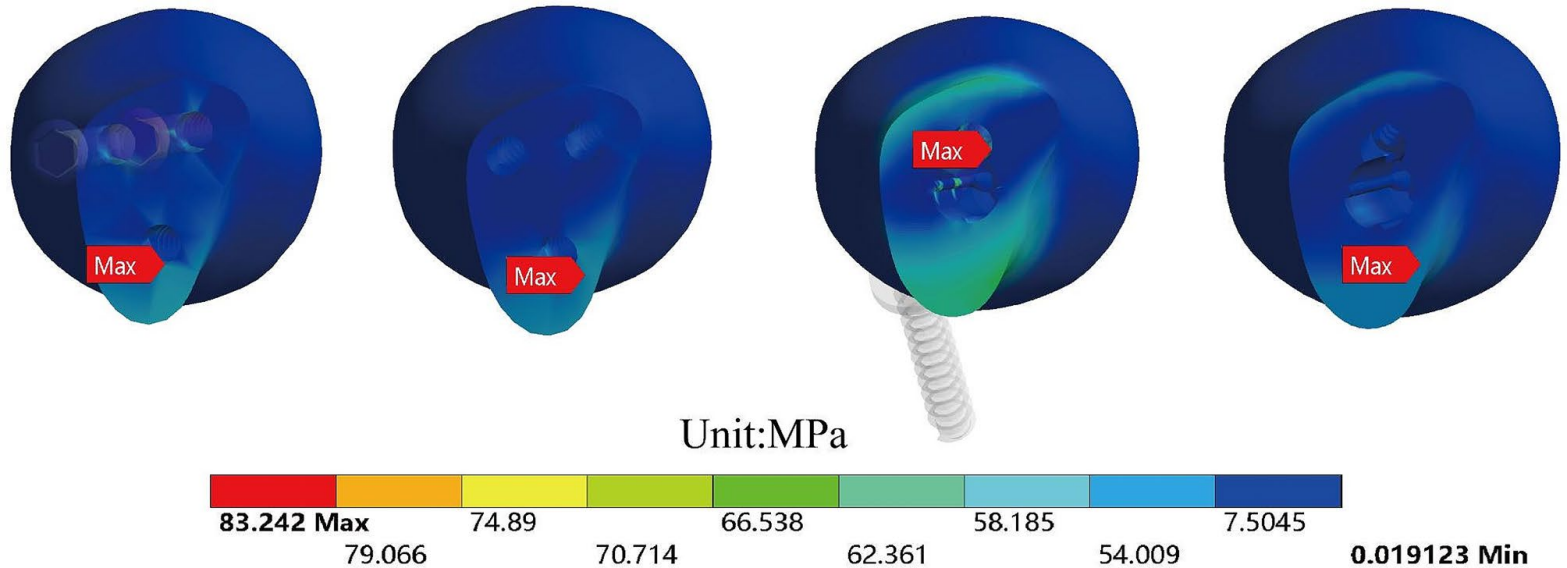
The equivalent stress of models: The transverse section of the femoral neck with preserved CSS (**A**); The transverse section of the femoral neck after removal of CSS (**B**); The transverse section of the femoral neck with preserved FNS (**C**); The transverse section of the femoral neck after removal of FNS (**D**)

**Fig. 7:**
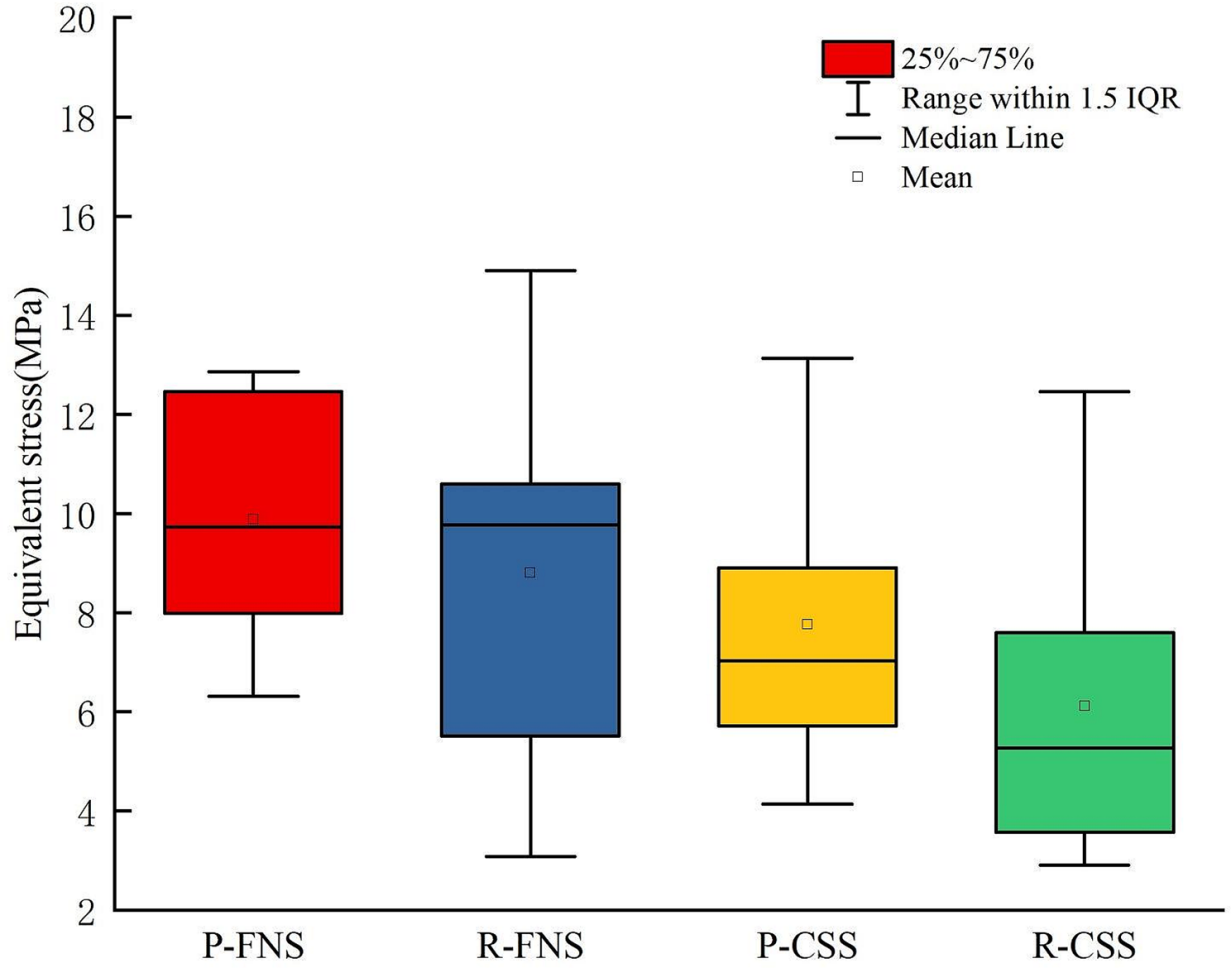
The equivalent stress on the reference plane of the femoral neck in various models: P-FNS (Preservation of FNS model); R-FNS (Removal of FNS model); P-CSS (Preservation of CSS model); R-CSS (Removal of CSS model)

The equivalent stress for each model was shown in Figs. 5 and 6. The red indicator bands represented stress peaks, while the ranges indicated stress concentrations. The equivalent stress for CSS and FNS were 55.21 MPa and 250.67 MPa, respectively. Compared to the removal of internal fixation, the model retaining internal fixation exhibited a significant increase in the stress range of the femoral neck. The stress shielding was more pronounced around the nail track. Figure 7 showed the equivalent stress on the cross-section of the femoral neck in each model after the removal of internal fixation. For comparison purposes, we selected the same cross-section of the femoral neck and calculated the average stress of the ten regions of interest. To better simulate real conditions, our reference plane was chosen as the Pauwels 60-degree angle of the femoral neck fracture plane. The average equivalent stress on the cross-section of the femoral neck before and after removal of CSS was 7.76 MPa and 6.11 MPa, respectively. The average equivalent stress on the cross-section of the femoral neck before and after removal of FNS was 9.89 MPa and 8.79 MPa, respectively.

## Discussion

FNS incorporates numerous advantages, including offering excellent angular stability, minimizing vascular damage, dynamic compression, and anti-rotation. At present, there have been numerous finite element analysis experiments [14, 19, 20] studying the immediate stability of FNS and CSS in treating femoral neck fractures. However, there is scarce mechanical analysis available regarding the effects post internal fixation removal. Due to the limited duration of FNS application and the lack of sufficient clinical cases, we conducted mechanical experiments to investigate the impact of FNS removal. Finite element analysis can simulate surgical procedures as well as the effects of different types of internal fixation devices in fracture treatment [2]. This helps optimize surgical plans and the design of internal fixation devices, improving surgical success rates and patient recovery outcomes. Through finite element analysis, different treatment options (such as surgery, conservative treatment, etc.) can be compared in terms of their impact on the healing process of femoral neck fractures. This assists doctors and researchers in selecting the optimal treatment strategies and predicting potential treatment outcomes [21].

In finite element analysis, total deformation refers to the comprehensive change in material shape resulting from external loading or modifications in boundary conditions. Total deformation helps evaluate the stability of a structure under external loading. When total deformation is small, the structure may be more stable, whereas larger deformations indicate potential risks of deformation or failure. Due to the simulated fracture healing model, no friction interface was set for the fracture. In contrast to the immediate stability advantage reported in previous literature for FNS treatment of femoral neck fractures [22], the total displacement of the model retaining FNS exceeded that of CSS. In essence, retaining internal fixation tended to provide the requisite stability, while the stability decreased after the removal of internal fixation. During the initial phase of fracture healing, the role of internal fixation is primarily to promote healing by providing stability. The internal fixation device immobilizes the fractured bones to prevent displacement and creates a stable environment conducive to healing. At this stage, the internal fixation typically needs to be sufficiently rigid and stable to support the fractured bones and minimize displacement. Once the fracture has healed, the role of the internal fixation device changes. At this stage, the same level of stability may no longer be necessary as the fracture has already healed. After fracture healing, if internal fixation devices remain in the body, they may lead to long-term issues such as metal fatigue, corrosion, allergic reactions due to electrolysis. These issues could potentially impact the long-term health and quality of life of patients.

Based on the results of equivalent stress, it appeared that retaining internal fixation may have led to notable stress concentration and stress shielding effects. From the color distribution of equivalent stress, it was evident that the area of stress concentration in FNS was significantly larger than that in CSS. In finite element analysis, equivalent stress is utilized to assess the material’s performance under complex loading conditions. The peak stress of FNS was five times greater than that of CSS. In theory, higher stress levels approach the material’s yield strength, posing a greater risk of fatigue. Additionally, retaining internal fixation increased the average equivalent stress on the cross-section of the femoral neck.

After the removal of internal fixation, the stress distribution in CSS channels became more dispersed, resulting in less impact on the proximal femur. Eberle’s research suggests that removing bone implants after healing is complete can restore bone strain to pre-fracture levels and may prevent further bone loss caused by stress shielding [23]. The stress shielding effect after internal fixation of femoral neck fractures refers to the phenomenon where the implanted plate or nails bear the load of the injured area post-surgery, relieving the bone itself from the same degree of stress. Consequently, the bone at the site of the femoral neck fracture may gradually weaken due to the lack of force stimulation. The human skeletal system is a dynamic tissue that responds to forces and loads. Under normal circumstances, the skeletal structure receives force stimulation that helps maintain bone density and strength. However, when internal fixation devices are used to stabilize fractures, they absorb and distribute the pressure at the injured site, reducing the forces exerted on the bone. Reduced stress at the fracture site may prolong the healing process. Insufficient force stimulation may lead to decreased bone density, increasing the risk of fracture recurrence. Muscles are crucial for maintaining skeletal stability and strength. Muscle atrophy due to lack of movement can also affect bone health.

In theory, after the removal of internal fixation for femoral neck fractures, the lack of support from hollow screws in the femoral head, combined with a decrease in bone density, would result in a significant reduction in the bone strength of the femoral head. To mitigate the stress shielding effect, bone grafting and postoperative rehabilitation may be typically recommended, including gradually increasing loads and exercise to restore bone and muscle strength and function.

Our study also has certain limitations. Firstly, our simulation focused on static analysis under single-leg standing conditions, without considering additional scenarios such as dynamic movements. Furthermore, our simulation was based on models of fractures that were well-aligned and healed, disregarding conditions such as malunion. Additionally, extensive clinical data and further mechanical analysis are imperative.

## Conclusions

The retention of internal fixation may contribute to improved stability of the proximal femur. However, there still existed risks of stress concentration in internal fixation and stress shielding in the proximal femur. Compared to CSS, the removal of FNS results in larger bone tunnels and insufficient model stability. Further clinical interventions are recommended to address this issue.

## Data Availability

The datasets used and/or analysed during the current study are available from the corresponding author on reasonable request.
